# The impact of COVID-19 on international development aid and health systems strengthening in low-income countries

**DOI:** 10.1016/j.amsu.2022.104772

**Published:** 2022-09-22

**Authors:** Emery Manirambona, Shuaibu Saidu Musa, Sarah Irakoze, Theogene Uwizeyimana, Dawa Gyeltshen, Deo Bizoza, Don Eliseo Lucero-Prisno III

**Affiliations:** College of Medicine and Health Sciences, University of Rwanda, Kigali, Rwanda; Department of Nursing Sciences, Ahmadu Bello University Zaria, Nigeria; Faculty of Medicine, University of Ngozi, Bujumbura, Burundi; Educational Development and Quality Center, University of Global Health Equity, Kigali, Rwanda; Eusa Hospital, Ministry of Health, Wangdue Phodrang, Bhutan; College of Medicine and Health Sciences, University of Rwanda, Kigali, Rwanda; Department of Global Health and Development, London School of Hygiene and Tropical Medicine, London, United Kingdom

**Keywords:** COVID-19, Global aid, Health financing, Health systems, LICs, Health equity

## Abstract

Health systems play a critical role in providing services that aim to improve, promote, restore, or maintain the health of communities. Unfortunately, health systems in low-income countries are fragile, having an adverse effect on the health of the population. Whereas international development aid remains crucial in strengthening health systems in low-income countries (LICs), COVID-19 has induced changes in the dynamics in the availability, provision and access to international development aid. These changes have aggravated the already weak health systems of LICs. Understanding the effects of the COVID-19 pandemic on the distribution of international development aid and how these effects impacted on the quality of the health systems in response to the outbreak is critical to improving the health of populations in LICs. This article discusses the impact of the challenges faced by LICs in the context of international development aid needed for the development of health systems.

## 1 Introduction

The World Bank releases the annual classification of the global economies which are divided into four categories—low-income countries (LICs), lower and middle-income countries (LMICs), upper-middle-income countries (UMICs), and high-income countries (HICs). The recent classification, calculated using the World Bank Atlas Method, classified 27 countries with $1045 or less in 2020 as LICs, with the majority (23/27) being in Sub-Saharan Africa [1]. This classification is based on gross national income (GNI) per capita in US dollar, which is defined depending on aspects such as “gross domestic product (GDP), plus net receipts from abroad of compensation of employees, property income and net taxes less subsidies on production” [2]. Factors influencing GNI are crucial to consider as they inform governments about the state of their economies, suggesting areas of improvement and allowing them to set effective policies for further economic growth.

The recent classification of the world's economies has shown major re-categorization of many countries. This is because of conflicts, wars, diseases, and other disasters that worsen the already low financial status of limited-resource countries. Significant in recent events is the COVID-19 pandemic which aggravated poverty worldwide and which weighed more heavily on countries whose income was already making them vulnerable LICs. The pandemic compounded poverty in different ways, leading over 119 million people into extreme poverty as of 2020 [3]. The worsened poverty during the COVID-19 outbreak has become more pronounced among LICs and has undoubtedly dampened the global health progress gained a few years ago.

Financial outcomes and health are inextricably linked, with the devastating impact of inadequate and low spending on health. To tackle the consequences of the economic burden on health, the UN and other global agencies prioritised financing LICs, yet there are challenges connected to providing this aid. Many of these global aid donors use a health system strengthening (HSS) approach to fund the poorest countries— through the HSS window or cross-cutting approach [4]. Health systems have a crucial role in providing services aimed at enhancing, promoting, restoring, or advancing the health of populations. A fragile health system hampers achieving the UN SDGs in general, including Universal Health Coverage (UHC). As such, international aid is crucial to strengthening health systems in LICs.

Therefore, comprehending the impact of the COVID-19 on international development aid and how it affected the strength of health systems is vital for evidence-based advocacy to donors and critical to enhancing the health of people in LICs. This article's aims are twofold: firstly, it explores the impact of COVID-19 on international development aid in LICs. Secondly, it argues the influence of these impacts on health systems strengthening in LICs. With particular focus on the six building blocks of health systems as explained by the WHO's health systems framework, which includes service delivery, health workforce, information, medical products (including vaccines, medications, digital equipment), financing, and leadership.

## 2 Methods

We reviewed data sources to determine the impact of international aid cuts due to COVID-19 on health systems in low-income countries. Using pre-determined search terms, a search on PubMed, PubMed Central, and Google Scholar was carried out. Data sources, i.e., pertinent English-language articles about aid cuts and difficulties in low-income nations, were the inclusion criteria. The references to the data sources were also examined to find pertinent data. Additional Data were gathered from statistics published, commentaries, policy briefs, and other news reports. Utilizing Mendeley Reference Manager, the collected articles were organised.

## 3 Results and discussion

The COVID-19 has weakened international economies globally but weighed more heavily on countries whose income was already making them vulnerable LICs. The economies in LICs were already fragile, and thus, the financial crisis caused by the pandemic has significantly affected the poorest parts of the world. Sub-Saharan Africa's economic growth has been expected to fall to less than 3.3% as of 2020 due to the COVID-19 pandemic, being the region's first recession in 25 years [5]. This was due to the disruption of activities that influence GDP on the continent. The disrupted activities that hampered the African economy include - but are not limited to – the slowdown in international trade, oil prices that dropped by half, and disruption of the supply chain of goods imported to Africa [6].

The disruption to the global economy caused by the COVID-19 pandemic brought with it challenges to the accessibility of crucial international development aid funds. Countries focused on their own survival and with key donors facing their own economic crises, funds allocated for global assistance were relocated to solve domestic issues [7]. International support to the LICs has fallen significantly as bilateral donors cut their aid. For instance, the UK Official Development Assistance (ODA) budget designated to support the COVID-19 response in the poorest countries was cut to more than 3.6 bn [7]. In addition, bilateral development aid was cut completely or there was disruption to grants that used to sustain some LICs.

The lack of funds has significantly resulted in a poor response to the pandemic and affected all building blocks of the health systems in the least developed world. Starting with healthcare services delivery: absence of sufficient funds resulted in an inadequate tracing and poor compliance to preventive measures as the poor population simply had to survive [8]. Similarly, the lack of funds affected the medical products supply chain including vaccines, medications, digital equipment, resulting in lack of personal protective equipment (PPE) such as masks and hygiene materials, lack of laboratory equipment which influenced lower testing capacity [8]. In addition, there has been limited availability of funding to train and enhance health professionals’ skills necessary during the outbreak. This inevitably may have hampered the abilities of the healthcare workforce - which has been disproportionately affected by the outbreak - to respond efficiently and effectively to the pandemic, resulting in poor health outcomes.

The international development aid cut has not only had adverse impact to COVID-19 response but also to other health program such as Disruption of cervical cancer screening [9]. Furthermore, as most of the vaccines are supported via international aid and imported from other countries, the LICs struggled to get financial support in order to book COVID-19 vaccines. More than half of the vaccine doses were already booked by wealthy countries [10]. Evidence of the disparity in the situation lies in the fact that LICs have yet to cover first dose delivery while HICs have been giving the booster doses. The late supply of vaccine support in LICs affected efficient service delivery and effective responsiveness.

Other implications of the lack of available funds in LICs are that these countries had to find ways to fill the HS financial gap caused by the COVID-19 pandemic to survive. In order to raise emergent funds to respond to the COVID-19 and its consequences, LICs had no option other than to accept loans given by HICs and private individuals, further straining the vulnerable economies [11]. Loans could potentially exacerbate the fragile health systems because most LICs are currently suffering the burden of loans, with their currency plummeting. Loans given to LICs have also the potential to become a future global health threat in these countries known to be at a higher risk of debt distress because it has been proved that these countries are unable to pay back [12]. Interestingly, most of the LICs were under “Heavily Indebted Poor Countries (HIPC)” as they had already the burden of debt-to-GDP ratio they cannot manage [6].

While health systems leadership in LICs devised policies to mitigate effects of the pandemic, some have faced management challenges due to the low funds available. Issues of transparency and accountability have been a concern among local leaders. Rampant corruption involving COVID-19 funds has been reported [13]. This lack of transparency impacts significantly on many health systems as it affects the quality of service delivery as well as information sharing imperative to responding efficiently to the pandemic [14]. Similarly, corruption can be the source of a lack of social and financial risk protection and becomes a public health concern with leaders driven by personal interest, often resulting in woefully fragile health systems in LICs that cannot appropriately manage disasters such as the COVID-19 pandemic.

A health system that functions well should ensure reliable and adequate data dissemination to mitigate the pandemic. However, the unavailability of funds in vulnerable economies has also affected health information systems. The vulnerable financial status could not set adequate internet, digital health equipment and infrastructure, along with electrical power, resulting in poor health information sharing and setbacks in epidemiological surveillance [15].

## 4 Conclusion

This article explored a few of the impacts the COVID-19 pandemic had on LICs. The pandemic has resulted in the lack of available funds from the international financial aid to respond to the COVID-19 in the LICs. This has unevenly impacted LICs’ health systems resulting in the poor response to the pandemic. The disparity in the COVID-19 response--vaccination coverage for example between the LICs and HICs is huge and this has the potential to prolong the pandemic. Therefore, it is vital for the international donors to support and prioritise health systems strengthening in LICs to end the pandemic sooner. Importantly, LICs should build an independent, resilient, and sustainable economy with independent health financing towards more robust health systems.

## 5 Recommendations

The COVID-19 pandemic has compounded the most vulnerable financial status in LICs. There is a need to boost and rebuild LICs. The UN and agencies worldwide must reconsider international development aid to support LICs development and strengthen the existing health systems. As such, improving the six-building blocks of the health system is crucial. Bolstering a resilient health service delivery is paramount to mitigating the pandemic. It is, therefore, crucial to improve accessible quality service delivery. Relevant to this, allocating funds to reinforce the workforce by training more health professionals, including community health workers, and engaging communities in the fight against this pandemic would increase workforce competence and achieve good health outcomes.

As the health information system plays a vital role in epidemiological surveillance, funds should be allocated to improve digital health and tract health services. Enhancing health information and data management is essential to alleviate the issues of transparency and accountability.

Furthermore, there is a need to enhance local production of medical products, vaccines, and other health technologies to ensure continuous access to them which are routinely inaccessible in low resource settings. Big pharma companies and global agencies should partner with local drug manufacturers or establish their branches in LICs in order to improve medicine and vaccines accessibility. This will improve health service delivery, the availability of medical products within an enabling digital health.

Loans should not be prioritised in this pandemic since many LICs are still struggling to recover from their social-economic situation, and the payback will be a significant burden. Instead, more efforts should be put into building good health system governance in LICs, establishing funding mechanisms and managing them transparently by ensuring that the population is prioritised. HICs and private donors should cancel all loans owed by LICs for the sake of health equity. Most importantly, it is also critical to build an independent economy in LICs in order to have strong and resilient health systems as this would be the only way to achieve the crucial Target 8 of Sustainable Development Goal 3 (SDG3)--Universal Health Coverage (UHC), which focuses on HSS.

## Ethical approval

The ethical approval is not applicable for this study.

## Source of funding

This research did not receive any specific grant from funding agencies in the public, commercial, or not-for- profit sectors.

## Author contribution

- Emery Manirambona conceived, designed and supervised the study, -Emery Manirambona, Shuaibu Saidu Musa, Sarah Irakoze, Theogene Uwizeyimana, Dawa Gyeltshen, Deo Bizoza^,^ Don Eliseo Lucero-Prisno III assisted in data collection, visualisation, writing and drafting the manuscript, - Don Eliseo Lucero-Prisno III reviewed, edited and proofread the manuscript with important intellectual additions, -All authors read and approved the final manuscript.

## Registration of research studies

1. Name of the registry: Not applicable
2. Unique Identifying number or registration ID: Not applicable
3. Hyperlink to your specific registration (must be publicly accessible and will be checked): Not applicable

## Guarantor

Emery Manirambona, Shuaibu Saidu Musa, Sarah Irakoze, Theogene Uwizeyimana, Dawa Gyeltshen, Deo Bizoza, Don Eliseo Lucero-Prisno III.

## Consent

Not applicable.

## Provenance and peer review

Not commissioned, externally peer reviewed.

## Declaration of competing interest

Authors have no conflicts of interest to declare.

## ABBREVIATION

LICs: Low-Income Countries
LMICs: Lower-Middle-Income Countries
UMICs: Upper-Middle-Income Countries
HICs: High-Income Countries
GNI: Gross National Income
GDP: Gross Domestic Product
COVID-19: Coronavirus Disease 2019
HSS: Health Systems Strengthening
HS: Health Systems
UHC: Universal Health Coverage
UN: United Nations
WHO: World Health Organization
SDGs: Sustainable Development Goal
ODA: Official Development Assistance
PPE: Personal Protective Equipment
HIPC: Heavily Indebted Poor Countries
